# Direct Substitution of Alcohols in Pure Water by Brønsted Acid Catalysis

**DOI:** 10.3390/molecules22040574

**Published:** 2017-04-01

**Authors:** Rosa Ortiz, Raquel P. Herrera

**Affiliations:** Laboratorio de Organocatálisis Asimétrica, Departamento de Química Orgánica, Instituto de Síntesis Química y Catálisis Homogénea (ISQCH) CSIC-Universidad de Zaragoza, C/Pedro Cerbuna 12, Zaragoza 50009, Spain

**Keywords:** alcohol, non-catalyzed, organocatalysis, S_N_1, water

## Abstract

With the increasing concern for sustainability, the use of environmentally friendly media to perform chemical processes has attracted the attention of many research groups. Among them, the use of water, as the unique solvent for reactions, is currently an active area of research. One process of particular interest is the direct nucleophilic substitution of an alcohol avoiding its preliminary transformation into a good leaving group, since one of the by-products in this approach would be water. The direct substitution of allylic, benzylic, and tertiary alcohols has been achieved through S_N_1-type reactions with catalytic amounts of Brønsted or Lewis acids; however, organic solvents are often required. In this review, the pioneering S_N_1 approaches performed in pure water and in the absence of a metal based Lewis acid are compiled and discussed.

## 1. Introduction

The direct nucleophilic substitution of an alcohol through an S_N_1-type reaction has been attracting chemists’ attention for many years [1], since one of the by-products in this process is water, and, due to the interest in trapping the resulting carbocation intermediate. For the activation of these S_N_1-type reactions using allylic, benzylic, and tertiary alcohols, a catalytic amount of a metal based Lewis acid is normally used [2,3,4,5,6]. In recent years, the organocatalytic version of this process has also received considerable attention [7,8].

Many efforts have been made by the group of Mayr to study the processes between carbocations and different nucleophiles, and an empirical equation to describe the reaction rate as a function of the absolute nucleophilicity (*N*) and the absolute electrophilicity (*E*) of the reactants has been developed [9,10]:

log*k*_20 °C_ = *S*_N_(*N* + *E*) (1)

**Equation 1**. Equation of the reaction rate as a function of the nucleophilicity (*N*) and the electrophilicity (*E*) [11,12].

Since water could also react as a nucleophile [13], the presence of water in an S_N_1-type reaction could compete with the real nucleophile of the process, hindering the attack of the latter on the electrophile. However, there are few pivotal examples that successfully demonstrate this challenging competitive process.

Moreover, the catalytic activation of alcohols is a difficult task since the hydroxy group is a poor leaving group and usually an excess of Brønsted acid or a stoichiometric amount of Lewis acid is required to achieve this goal [14,15,16]. The development of catalytic methods using Brønsted or Lewis acids is therefore highly desirable. However, the use of water as a reaction medium presents many advantages from the economical, environmental and safety [17] point of view and also in terms of the reactivity and selectivity [18] of the processes developed in this medium.

In the continuous search for a more environmentally friendly medium, the use of water as solvent or as co-solvent for reactions has received considerable attention in recent years [19]. Due to the poor solubility of organic compounds in water or the high reactivity that some of them display in this medium, the use of aqueous mixtures in organic synthesis was avoided for many years. However, its attractive practical advantages over other organic solvents have increased the interest in performing new processes in this reaction medium [20,21,22,23,24,25,26,27,28]. 

In this review, we aim to compile and discuss the scarce examples out of attempts to trap the carbocation generated from an alcohol using pure water and in the absence of a metal Lewis acid. The term “on water” or “in water” will be used depending on the authors’ assignment. Based on the classification of Sharpless, the reactions are named as “on water” protocols when some of the reactants are insoluble in the aqueous medium [29,30,31,32,33,34,35]. It is important to note that a few metal-catalyzed examples have been also developed “on water”, such as with gold(III) [36,37], In(III) [38] or Pd(0) [39,40,41,42,43]. However, high temperature or the use of a co-catalyst or an acid additive is necessary. 

## 2. Nucleophilic Substitution of Alcohols in Pure Water

### Non Chiral Brønsted Acid Catalysts and Non-Catalyzed Examples

In 2007, Kobayashi and co-workers developed a pioneering protocol for the dehydrative nucleophilic substitution of benzyl alcohols using different nucleophiles and employing DBSA (dodecylbenzenesulfonic acid) as a surfactant-type Brønsted acid catalyst in pure water [44]. 

Based on previous results from the same research group using DBSA to catalyze the dehydrative esterification of carboxylic acids with alcohols and etherification of alcohols in water [45,46,47], the authors envisioned the possibility of using the same catalyst to promote the nucleophilic substitution of alcohols in water.

In the preliminary screening of the reaction model, a Friedel–Crafts-type substitution reaction of benzhydrol with 1-methylindole, common Brønsted acids such as AcOH (0% yield), TFA (3% yield), TfOH (8% yield), TsOH (3% yield) or even a long chain carboxylic acid (C_9_H_19_COOH, 0% yield) were not effective catalyzing the process. In contrast, DBSA catalyzed the reaction giving promising results (85% yield). Both the surfactant property and the strong acidity of DBSA were found to be crucial for the success of the process.

**Scheme 1:**
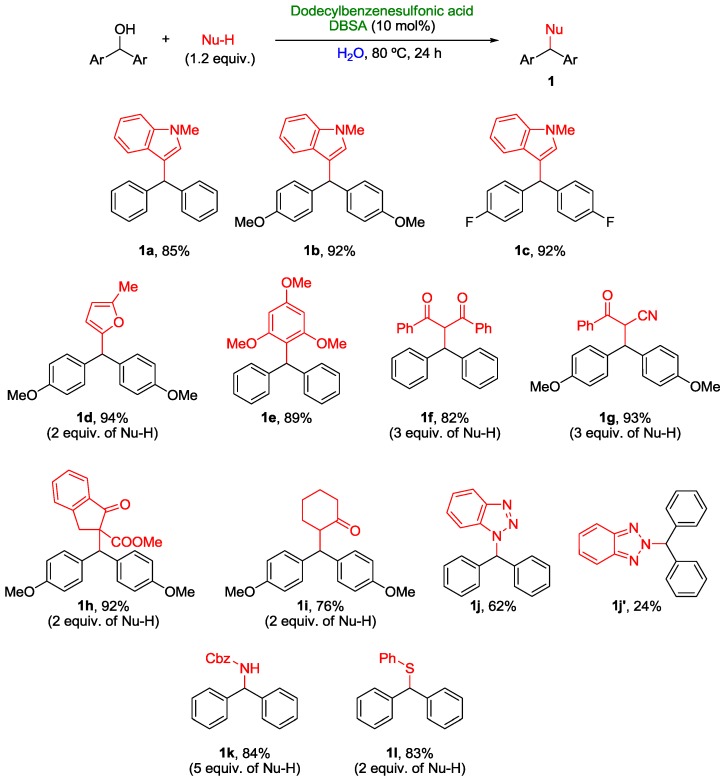
Scope of the substitution reaction of different benzhydrol derivatives.

The scope of the reaction was extended to a variety of nucleophiles in their reaction with different benzhydrol derivatives (Scheme 1).

Benzhydrols with electron-donating or electron-withdrawing groups reacted with electron-rich heteroaromatic or aromatic compounds to afford compounds **1a**–**e** in very good yields. When compounds with α-acidic protons were used, the addition also proceeded smoothly with high yields (**1f**–**i**). The reaction can be applied to form satisfactorily C-N and C-S bounds, as demonstrated by the formation of products **1j**–**1l**.

**Scheme 2:**
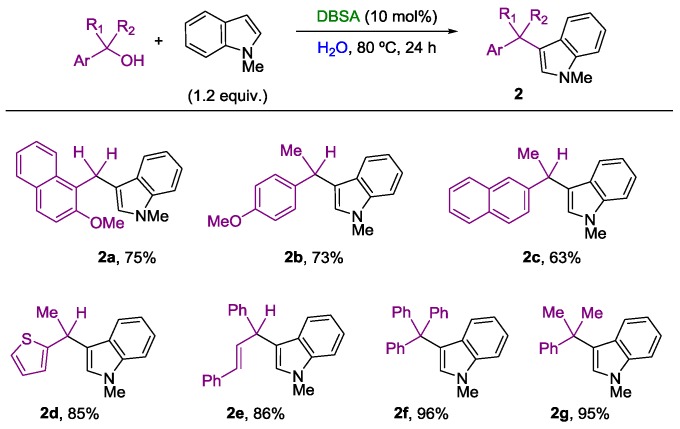
Scope of the substitution reaction using different primary, secondary and tertiary benzyl alcohols.

The substrate scope was also tested (Scheme 2). Thus, primary, secondary and tertiary benzyl alcohols were successfully employed to obtain **2a**–**g** in moderate to good yields. Substrates containing heteroaromatic and allylic alcohols also worked in this approach (**2d** and **2e**).

**Scheme 3:**
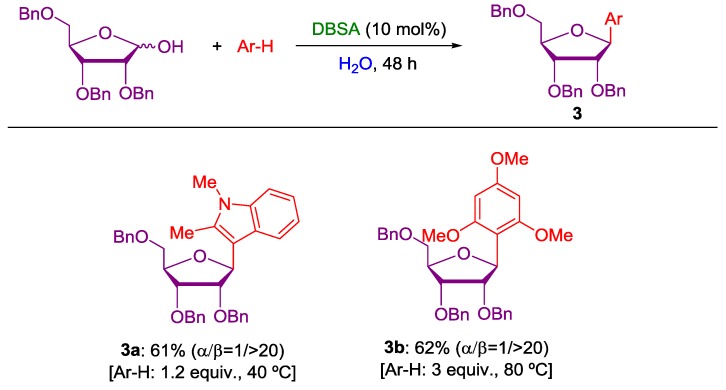
Selective dehydrative C-glycosylations of 1-hydroxy sugars in water.

Finally, the authors explored an additional application for the developed method. The selective dehydrative C-glycosylation of 1-hydroxy sugars in water was addressed, since methods for the synthesis of C-glycosides starting from 1-hydroxy sugars using catalytic amounts of an activator were scarce (Scheme 3). The reaction between 1-hydroxy-d-ribofuranose with electron-rich heteroaromatic or aromatic compounds proceeded smoothly to afford **3a** and **3b** in good yields and excellent β-selectivity.

**Scheme 4:**
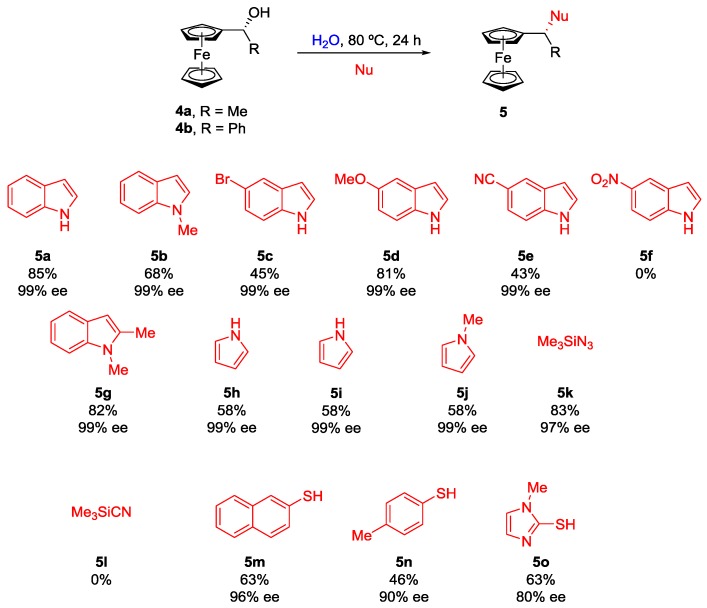
Scope of nucleophilic substitution of chiral ferrocenyl alcohols “on water”.

Cozzi and co-workers had studied the nucleophilic substitution of ferrocenyl alcohols enantiomerically enriched in organic solvents and with catalytic amounts of indium salts [48]. Based on these results, the same group explored the viability of the nucleophilic substitution of chiral ferrocenyl alcohols “on water”, without the presence of Lewis acids, Brønsted acids, surfactants or any other catalyst (Scheme 4) [49].

The authors tested different nucleophiles in the reaction model depicted in Scheme 4. Based on the results, a few points can be made. The conversions observed with indoles bearing electron-withdrawing substituents (**5c**, **5e**, **5f**) were lower than with the other nucleophiles. In the case of the product obtained from nucleophile **5i**, the reaction must be carried out with 5–10 equiv. of pyrrole (interestingly, the absence of Lewis acids avoided its polymerization). Acidic nucleophiles (i.e., 4-nitrophenol) or nucleophiles able to be hydrolyzed in water to weak acids (Me_3_SiCN to HCN) promoted the reaction of ferrocenyl alcohol with itself.

It is likely the stability and the easy formation of the ferrocenyl cation that probably play an important role in the efficiency of this reaction. These results are in agreement with the reactivity of indoles predicted by Mayr’s nucleophilicity scale [50], where many electron-rich π-systems are more nucleophilic than aqueous acetone or aqueous acetonitrile; hence, in slightly basic or neutral conditions, the carbocation intermediate generated in an S_N_1-type reaction could be trapped with electron-rich π-systems. This developed example, using mild reaction conditions, water, and, in the absence of co-solvents, additives, Lewis, or Brønsted acids, could be used to introduce the ferrocene moiety into biological molecules [51].

**Scheme 5:**
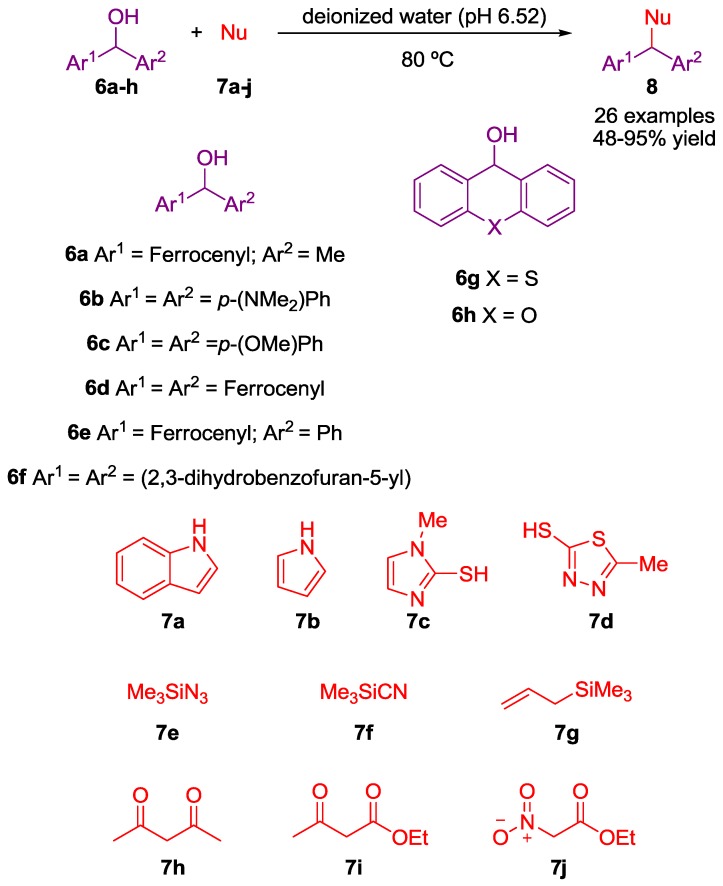
Scope of nucleophilic substitution of alcohols “on water”.

The same research group later extended the investigation to other alcohols and nucleophiles performing the reaction “on water” in the absence of a catalyst (Scheme 5) [52]. The final products **8** were obtained with moderate to very good yields.

The direct generation of carbocations on water from alcohols is probably driven by the formation of hydrogen bonds between water and the hydroxy group of the alcohol. This assessment is based on the Marcus and Jung proposal, according to which the formation of hydrogen bonds on the interface between water and oil is responsible for the acceleration of the reactions “on water” [53].

**Scheme 6:**
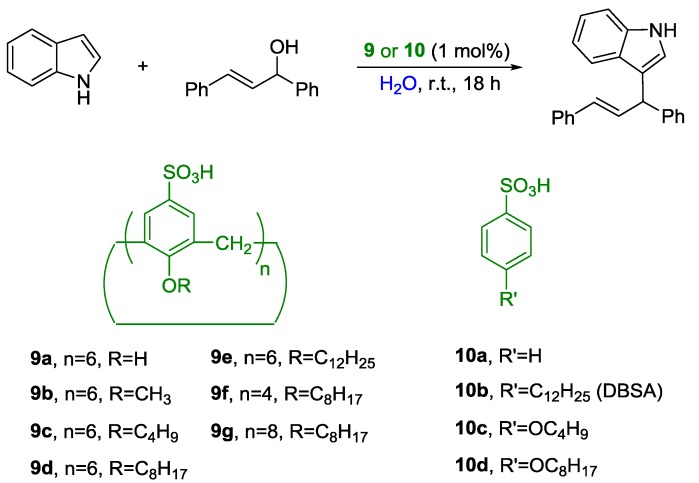
Screening of calix[*n*]arene and sulfonic acid derivatives.

Liu, Wang and co-workers developed in 2008 a methodology that includes the use of calix[*n*]arene sulfonic acids bearing pendant aliphatic chains as surfactant-type Brønsted acid catalysts for allylic alkylation with allyl alcohols in water [54]. The calix[*n*]arene sulfonic acid, which was prepared by the Shinkai protocol, can be considered as a series of *p*-alkylbenzenesulfonic acids linked by methylene spacers and provides a hydrophobic cavity [55]. The screening of the catalysts in the reaction model depicted in Scheme 6, afforded catalyst **9d** as the catalyst of choice (83% yield). This good result was attributed to the formation of micelles in the water. The small value of the parameter cmc (critical micelle concentration) for catalysts **9c**–**g** indicates that a small amount of the catalyst is enough to form a micelle in water (for **9a** and **9b**, the value of cmc was not calculated). However, with **9e**, the yield decreased to 61%, probably due to the formation of unimolecular micelles rather than molecular aggregates (Shinkai’s suggestion). The size of the cavity had no effect on the yield (**9d** 83% and **9f** 82%) up to a point because when **9g** was used, the yield decreased slightly (74%) probably due to the formation of a larger cavity.

For comparative reasons, catalysts **10a**–**d** were also explored. With **10a** (TsOH), the yield was very low (10%). The use of a polystyrene-supported sulfonic acid led to a 49% yield, but when surfactant-type Brønsted acids **10b** and **10d** were used, the results improved significantly (77 and 80%, respectively). However, comparing **9c** and **10c**, which have the same alkyl group, **9c** exhibited higher catalytic activity, giving 74% yield vs. 45% obtained with **10c**. Based on these results, it seems that the formation of micelles in water using surfactant-type catalysts is crucial for the catalytic alkylation reaction of allyl alcohols in water.

**Scheme 7:**
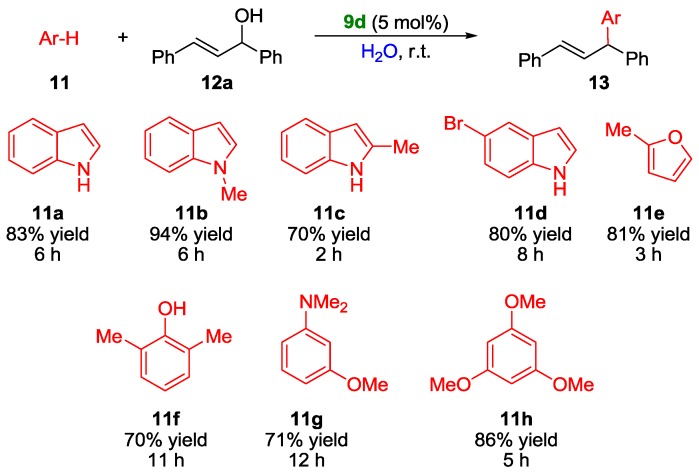
Scope of the allylic alkylation reaction of **12a**.

With the best conditions in hand, the authors explored the scope of the reaction using different aromatic compounds **11** (Scheme 7).

**Scheme 8:**
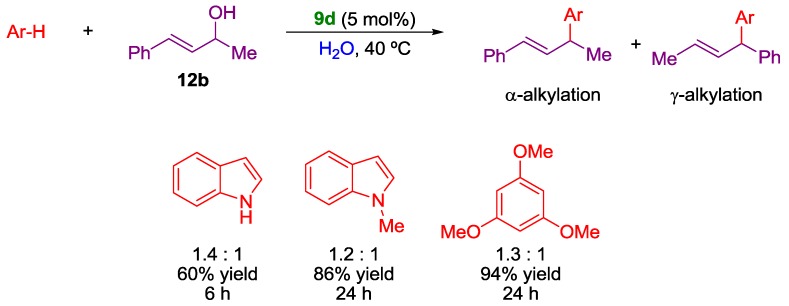
Scope of the allylic alkylation reaction of **12b**.

When the less reactive allylic alcohol **12b** was tested, the reaction was carried out at 40 °C in order to improve the yield and the regioselectivity of the process, although this was very poor (Scheme 8).

A recycling experiment was carried out using the model reaction depicted in Scheme 6. Thus, when catalysts **10b** or **10d** were used, the second run gave no product. However, when **9d** was used as the catalyst, the reaction produced the product at a high yield (92%–96%) even after seven cycles.

To extend the scope of the calixarene-SO_3_H as catalysts in water, the Friedel–Crafts alkylation with benzyl and propargyl alcohols was also performed but at 80 °C, giving very good results.

**Scheme 9:**
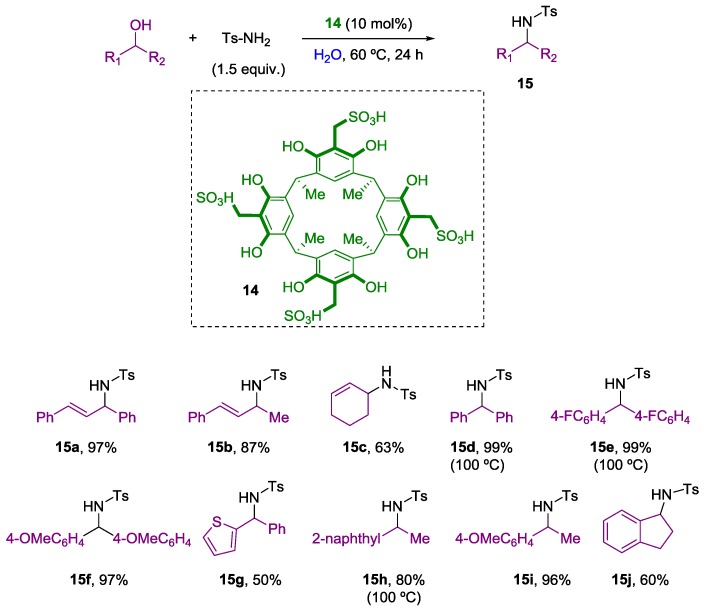
Dehydrative amination of alcohols in pure water.

Although different methods for the catalytic amination of alcohols using metal catalysis in organic solvent systems have recently been reported, the elimination of hazardous solvents and the introduction of water are desirable. In this context, Shimizu and co-workers developed the water-soluble calix[4]resorcinarene sulfonic acid **14** and used it as an efficient reusable catalyst for three-component Mannich-type reactions in water [56]. Based on this precedent, a new dehydrative amination of alcohols in water was developed by the same research group (Scheme 9) [57], using **14** as a Brønsted acid catalyst. 

Catalyst **14** was the best choice among the variety of Brønsted acid catalysts examined. The scope of the reaction was explored with the use of different allyl and benzyl alcohols. Both acyclic and cyclic allyl alcohols worked in this process giving rise to products **15a**, **15b**, **15c**. Amination of benzyl alcohols bearing electron-donating or electron-withdrawing groups proceeded cleanly (**15d**–**15j**), and heteroaromatic alcohols could also be used (**15g**).

**Scheme 10:**
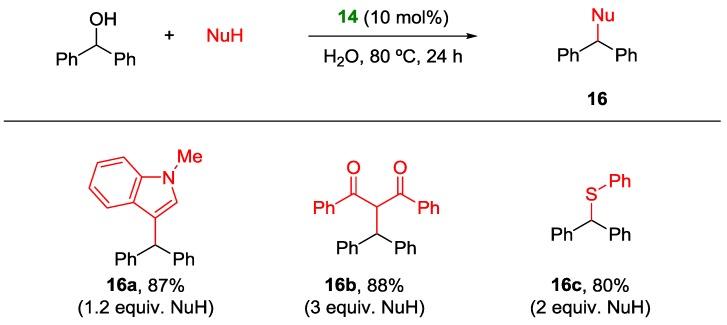
Scope of different nucleophiles.

The reaction with other nucleophiles was also tested and afforded good results for three different substrates (products **16a**, **16b** and **16c**) (Scheme 10).

The reusability of the catalyst **14** was studied after each reaction, extracting the product with EtOAc and recovering the aqueous solution with the catalyst for the next cycle. Even after five runs, the yields of the reactions were practically unchanged (92%–95% yield).

**Scheme 11:**
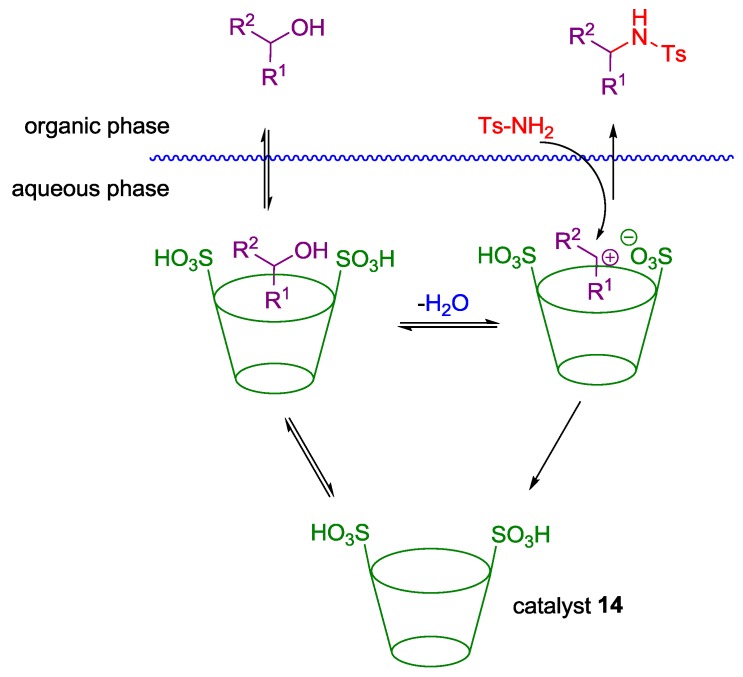
Possible reaction mechanism.

Based on the experimental results, a plausible mechanism was proposed. First, the catalyst **14** would form a host–guest complex with the alcohol in the interphase layer. Then, the dehydration reaction would be promoted by the sulfonic acid groups present in the structure of **14**, and the resulting cation would undergo nucleophilic attack, affording the products and regenerating the catalyst (Scheme 11).

**Scheme 12:**
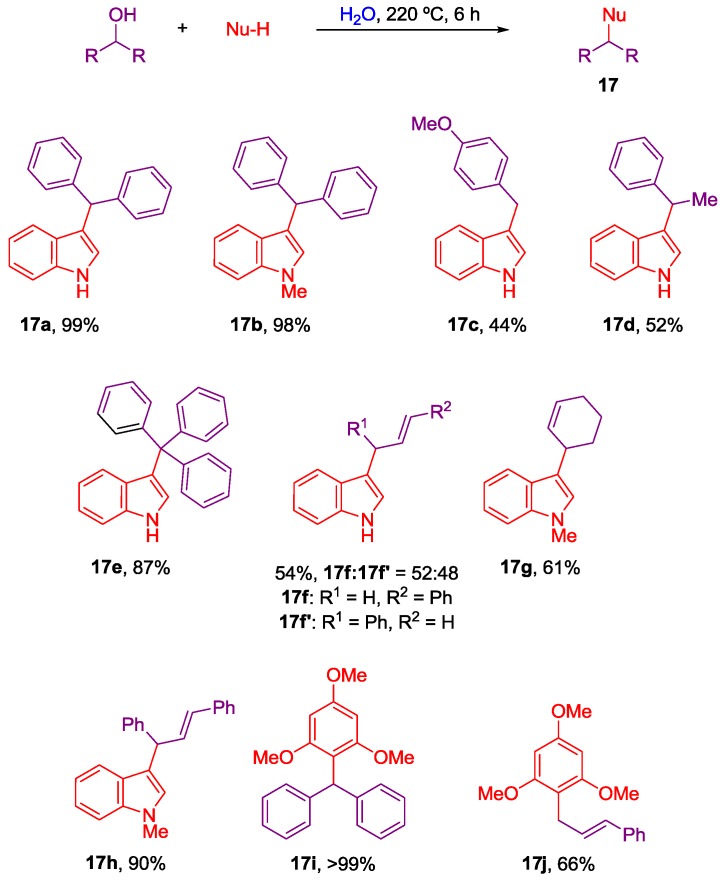
Allylic and benzylic substitution reaction in high-temperature water.

More recently, Hirashita and co-workers developed a new example using water in an autoclave at high temperature (220 °C) to trap the carbocation from a variety of benzyl and allylic alcohols with 1,3-dicarbonyl compounds and activated aromatic compounds. The desired alkylated products **17** were obtained with moderate to high yields in the absence of catalysts (Scheme 12) [58].

It is remarkable, that the appropriate substrates for this reaction were limited to electron-rich aromatic compounds, indicating that the transient intermediates are not electrophilic and/or stable enough under these conditions to promote the alkylation process. 

## 3. Conclusions

The direct nucleophilic substitution of an alcohol through an S_N_1-type reaction has been a challenge for many research groups for many years. The interest in this reaction has increased due to the fact that water is the only by-product in this process and because the resulting carbocation intermediates can be trapped. In this review, we have covered the scarce examples described to trap the reactive carbocation generated in pure water using an organocatalytic Brønsted acid or in the absence of a catalyst. Even when water could also react as a nucleophile in an S_N_1-type reaction competing with the real nucleophiles of the processes, the selected examples discussed in this review demonstrate the viability and success of this process. Different allylic, benzylic and tertiary alcohols have been used with a broad variety of nucleophiles and the corresponding carbocations have been successfully trapped using water with high yields. Sulfonic acid derivatives used as surfactants or calixarenes catalysts have been employed to promote this methodology. 

Although there are still scarce examples of the organocatalytic activation of alcohols in aqueous phase [59], as the unique solvent for the reactions, with the continuous search for environmentally friendly reaction media, we presume further progress will be made in this stimulating field and many other interesting and green approaches are expected in the near future.
